# Extended Reality to Assess Short-Term Spatial Memory—A Comparative Study of Mixed Reality, Augmented Reality, and Virtual Reality

**DOI:** 10.3390/s24247938

**Published:** 2024-12-12

**Authors:** David Ponce, Magdalena Mendez-Lopez, Javier Lluch, M.-Carmen Juan

**Affiliations:** 1 Instituto Universitario de Automática e Informática Industrial, Universitat Politècnica de València, C/Camino de Vera, s/n, 46022 Valencia, Spain; daponse@doctor.upv.es (D.P.); jlluch@dsic.upv.es (J.L.); 2 Departamento de Psicología y Sociología, Universidad de Zaragoza, 50009 Zaragoza, Spain; mmendez@unizar.es; 3 IIS Aragón, 50009 Zaragoza, Spain

**Keywords:** mixed reality, augmented reality, virtual reality, short-term memory, spatial memory, optical see through, HoloLens 2, assessment, user experience, user performance

## Abstract

A Mixed Reality (MR) application using an optical see-through headset was developed to assess short-term spatial memory. A study with 29 participants was conducted. Data from this study were compared to two previous studies using mobile Augmented Reality (AR) and Virtual Reality (VR) with headsets. When comparing the three technologies (MR, AR, VR) for the performance variables, there were no statistically significant differences for either the total number of correctly placed objects or the total number of attempts. However, the MR application required more time than the AR and VR applications in the evaluation phase and more time than the VR application in the learning phase. Our arguments for the longer time are the novelty of the MR application for the participants and the characteristics of the applications. The key results from the MR study include the following: (1) the objects used in the MR application were correctly positioned on a map, which implies that the memory acquired with the MR application is effectively transferred to the user’s mental map; (2) for the performance variables, there were no significant differences in the results by gender; (3) and the usability rating decreased with computer experience. The results show that the MR application is effective for spatial memory assessment and was well rated by the participants. The three technologies, along with suitable hardware, are effective for spatial memory assessment. However, MR using optical see-through headsets offers advantages over mobile AR and VR using headsets, discussed in this publication.

## 1. Introduction

Spatial-location memory is a type of declarative memory for spatial information [1,2]. This memory allows people to make precise associations between objects and their spatial locations. Spatial-location memory in large environments allows people to remember the locations of small objects that typically change their location in the environment (e.g., personal items) and is essential for success in typical daily activities at home and at work [3]. Short-term spatial memory is defined as the ability of an individual to remember the location of items in the environment for short periods of time [4]. Humans, like most animals, use it for tasks such as orienting ourselves in space, remembering a path, or remembering where we have left our belongings.

Conventional assessments of short-term spatial memory typically involve the presentation of objects on paper or screens [5,6] while participants are seated. However, previous works have highlighted the importance of physical movement in the acquisition of spatial skills [7]. Virtual Reality (VR), Augmented Reality (AR), and Mixed Reality (MR) are technologies that can be exploited to develop tools for studying spatial memory.

Extended Reality (XR) is an umbrella term for AR, MR, and VR [8]. Milgram and Kishino’s continuum [9] is a widely recognized classification framework that classifies AR, VR, and MR technologies along the ‘virtuality continuum’ ranging from completely real environments to fully virtual ones. In AR, the real environment is ‘augmented’ by integrating virtual objects [9]. A more exhaustive definition was provided by Azuma [10]. He describes AR as systems that possess three key characteristics: they combine real and virtual; they are interactive in real time; and they are registered in 3-D. In VR, the user is fully immersed and can interact with an entirely artificial world [9]. In MR, real-world and virtual objects coexist, lying anywhere between the extremes of the ‘virtuality continuum’ [9]. It is important to highlight that the ‘virtuality continuum’ was explicitly concerned with only visual displays and was established 30 years ago. Attempts to expand upon Milgram and Kishino’s continuum have focused on exploring the boundaries between physical and virtual spaces in MR environments [11], proposing a classification framework for multisensory experiences [12], introducing new concepts such as mediated reality [13], proposing a conceptual framework for MR [14], or revisiting Milgram and Kishino’s continuum [15].

There is no clear, universally accepted definition of what MR is or how it differs from other concepts. Sometimes, MR and AR are used interchangeably [14]. In addition to the above definition of MR, Skarbez et al. [15] defined MR as an environment that seamlessly combines elements from the real and virtual worlds into a unified perceptual experience. In such an environment, users simultaneously perceive real and virtual content, often across multiple senses. According to Parveau and Adda [16], MR is a paradigm that integrates technologies that are capable of mapping the user’s environment to present 3D virtual content registered in space and time. Virtual elements can be spatially aligned with the physical world, the user, or other virtual or real objects. Moreover, the MR experience should prioritize the user, providing natural and responsive interactions. One commonly recognized definition describes MR as a blend of real and virtual environments that creates an immersive experience, enabling interaction among physical and digital objects [8,17]. In the case of our application with HoloLens 2 (Microsoft, Redmond, WA, USA), we categorized it as MR because, in our opinion, it best fits the above definitions of MR, although our application could be classified as AR, as it also meets the criteria for such a classification.

XR technologies are particularly useful when users need to navigate real-world environments physically, as they offer experiences that closely mimic real-world tasks. Traditionally, VR has not allowed the user to physically move to navigate the virtual environment, and navigation is conducted using controllers (e.g., joysticks). However, physical navigation became possible with the advent of standalone VR headsets such as Meta Quest (similar or superior). AR and MR allow the user to physically move around the real environment. In addition, the latest MR headsets allow the user to have a natural perception of the real environment, a full blending of the virtual elements, and a natural interaction between virtual and real elements. In our case, we used HoloLens 2. HoloLens 2 is a standalone headset that does not need to be connected to any other device to work, giving the user complete freedom of movement. The digital elements blend into the real environment giving a sense of integration. Interaction is gestural with the hands, and no controllers are required. Voice interaction is also possible.

In this work, we present a new MR application that runs on an MR headset (HoloLens 2) for the assessment of short-term spatial memory. Our MR application does not require any physical elements to be added to the scene for tracking, and participants are free to move around indoor spaces. The application task is divided into three phases: a familiarization phase, a learning phase, and an evaluation phase. In the familiarization phase, the participants become familiar with the headset and the application. In the learning phase, the participants search for virtual 3D objects distributed in a physical room. In the evaluation phase, the participants recall these objects and use the MR application to correctly place the objects in the room. The application stores useful data for further study (e.g., the successes and errors, the Learning Time, the Evaluation Time). The main objective is to prove whether MR is useful for assessing short-term spatial memory and has advantages over AR and VR. A study was conducted to validate our MR application. Our study involved twenty-nine participants and aimed to compare the data with two previous studies, one that used AR on a mobile device [18] and another one that used a virtual environment that was the modeled room in which the AR and MR applications were validated [19]. The main hypothesis of this work is that MR running on headsets such as HoloLens 2 or superior is an effective technology for assessing short-term spatial memory and that the experience is satisfactory for the participants.

### 1.1. Spatial Memory

Short-term memory and long-term memory have been studied extensively in animals [20]. One of the classic experiments is to create mazes with rewards whose path must be memorized in order to find the reward more quickly [21]. Similar studies have also been conducted in humans [22]. Other methods that have been used to assess spatial memory in humans involve graphic representations or images on paper [23,24] or on a screen while the subject remains seated in a chair, but these methods do not involve physical displacement [25,26,27].

Using visualization devices, new methods have been developed to assess spatial memory in humans by simulating environments or large rooms without the need to use real rooms. For example, Shore et al. [28] used computer-generated virtual environments to study spatial short-term memory in humans. The subjects were able to move around a large virtual space displayed on a screen using a keyboard or a joystick.

According to previous works [29,30], there is a neural process in humans that is responsible for updating the mental map of the environment when the subject physically moves, which does not occur when the objects in the environment are the ones that move. In these studies, a subject was shown a series of objects around a round table. After the objects were hidden, sometimes the subject moved around the table, and sometimes the table rotated. The users were able to recognize and remember the position of the objects much better when they were the ones moving around the table, rather than when the table rotated. The results suggest that subjects recognize and remember better when they are the ones moving. This supports the hypothesis that people are more adept at remembering spatial landmarks when they are actively engaged in physical movement. Therefore, AR and MR offer potential advantages for the development of applications that allow physical navigation through an environment. AR and MR applications that encourage user movement may have a positive impact on memory tasks compared to other methods in which the subject remains static or seated.

### 1.2. Technology-Assisted Assessment of Spatial Memory

Short-term spatial memory can be assessed using paper-and-pencil tests [23,24]. The first computerized assessment tools used the same principles, but they replaced paper with a screen [31]. This already offers some advantages over analog tools, such as the possibility of collecting some variables in an automated way (successes, failures, and reaction times).

The incorporation of VR, and later AR, has opened up new possibilities for the assessment of spatial memory. These technologies allow the user’s interaction with objects to be natural, and navigation through the environment can be achieved with the user’s physical displacement. In addition, there is no need to model the environment in AR since the user is in direct contact with the real world. Experiences with these technologies are much more similar to everyday activities. The first applications of virtual environments to assess spatial memory used a monitor as the display device, and the subject used a keyboard, joystick, or mouse to navigate and interact with the environment while remaining seated [25,26,27]. Later studies used the physical displacement of the subject to explore the virtual environment [32,33]. The use of AR is more recent. The first mobile AR applications [34,35] used images as targets for tracking and thereby to determine the position and orientation of virtual objects in the real environment. More recent works have introduced mobile AR applications based on Simultaneous Localization and Mapping (SLAM) [36,37], eliminating the need for physical images in the real world. Other authors have developed an MR application that incorporates holographic grids to study their impact on distance estimation and location memory [38]. Their results suggested that the display of a grid led to more accurate distance estimates, but location memory performance was worse. Auditory stimuli have also been used to assess spatial memory [39]. A mobile AR application containing both visual and auditory stimuli was developed to assess spatial memory [39]. Their study aimed to compare the participants’ performance between visual and auditory stimuli and found similar success rates, but memory for spatial–visual associations was dominant since the spatial location of visual stimuli was remembered more precisely and rapidly. Tactile stimuli have also been studied for spatial memory assessment [18]. The results showed similar success rates between visual and tactile stimuli, but again, memory for spatial locations was more precise and rapid with visual stimuli [18].

## 2. Materials and Methods

### 2.1. Participants

This study, carried out using an MR application, involved a total of 29 participants between the ages of 20 and 62 (mean ± SD: 32.24 ± 10.39), of whom 14 (48.27%) were men and 15 (51.72%) were women. Data from this study were compared with two previous studies [18,19]. The first study [18] used a mobile AR application with 47 participants (age, mean ± SD: 30.98 ± 9.72), of whom 70% were men and 30% were women. The second study [19] used a VR application with 25 participants (age, mean ± SD: 26.28 ± 13.33), of whom 52% were women and 48% were men. The participants of the three studies belonged to distinct, non-overlapping groups. Accordingly, the analysis was structured as a between-groups comparison to examine the differences among these separate populations. The participants of the MR study were informed about the procedure and the objectives of this study prior to their participation and gave their consent to participate in this study. The MR study was approved by the Research Ethics Committee of the Universitat Politècnica de València, Spain, and was conducted in accordance with the Declaration of Helsinki.

### 2.2. Measures

#### 2.2.1. Performance Variables

Performance variables were automatically stored by the application during its use. The variables used for statistical analysis in this study are as follows:

- Total Objects: The total number of objects correctly placed at the end of the evaluation phase. The minimum is 0, and the maximum is 8 if the participant placed all of the objects correctly. The application considers an object to be correctly placed if it is in the correct location with a margin of error of approximately 50 cm.
- Total Attempts: The total number of failed attempts. This variable stores the total number of failed attempts to place an object by the user, summing the number of failures for each object. The minimum is 0, and the maximum is 24 (3 for each of the 8 objects).
- Learning Time: The time (in seconds) that the user needs to memorize the positions of the objects. The users must touch each of the objects with their hand when they think that they have memorized its position. When the user touches the last object, the application registers the time spent. The order in which the objects are memorized is not taken into account. The order is at the user’s discretion.
- Evaluation Time: The time (in seconds) taken by the user to place all of the objects in their positions during the evaluation phase. The time is automatically saved when the participant places the last object or fails for the third time.

For each task, all of these data are automatically associated with an anonymous user identifier that is used to link the task performance variables with the map task performance variables and the subjective variables obtained through the questionnaire.

#### 2.2.2. Other Performance Variables

In addition to the variables stored by the application, a performance task was also carried out, i.e., a map task from which the following variable is obtained.

Map task: The number of objects correctly placed on a 2D map representing the same room in which the MR task was performed. The participants draw the position of the objects that they remember from the MR task on a map.

#### 2.2.3. Subjective Variables

The participants filled out a questionnaire with 75 questions from which demographic data and subjective data were obtained. The subjective data were grouped into 15 variables for statistical analysis: no cybersickness, enjoyment, concentration, usability, competence, calmness, expertise, non-mental effort, non-physical effort, ergonomics, satisfaction, presence, anxiety, experience with computers, and video game experience. To obtain the values of these variables, the values of the answers to the questions related to the concepts in the form were grouped, and their arithmetic mean was obtained. All of the questions were formulated in a positive way. Most of the questions in the questionnaire were designed to be answered on a Likert scale with values ranging from 1 (totally disagree) to 7 (totally agree). The questions related to anxiety had values which ranged from 1 (not at all) to 3 (very much). The questions about computer and video game experience had values which ranged from 1 (not at all) to 5 (very much). In addition to the explicit question about video game experience, the participants were asked about their video game experience by genre (divided into five groups): first person, simulators, strategy, turn-based, and role-playing. These questions asked about the number of hours per week the participants played when they played the most, with six possible answers: never; >0 to 1; >1 to 3; >3 to 5; >5 to 10; and >10. The questionnaire was designed specifically for this study, although some questions are adaptations of commonly used questionnaires [40,41,42].

### 2.3. Procedure

The participants were selected randomly, with an attempt to maintain a balanced proportion of men and women and to meet the requirement of being adults aged 18 or older. Prior to the start of this study, the steps and the objectives of this study, how the collected data would be processed, and the basic instructions on how to use the HoloLens 2 headset were explained to the participants. From this point on, the participants would complete the MR task, then the map task, and finally the questionnaires.

#### 2.3.1. MR Task

In this section, the application and the hardware and software used are introduced, and then the task itself is described in detail.

*The Mixed Reality application*

**Figure 1:**
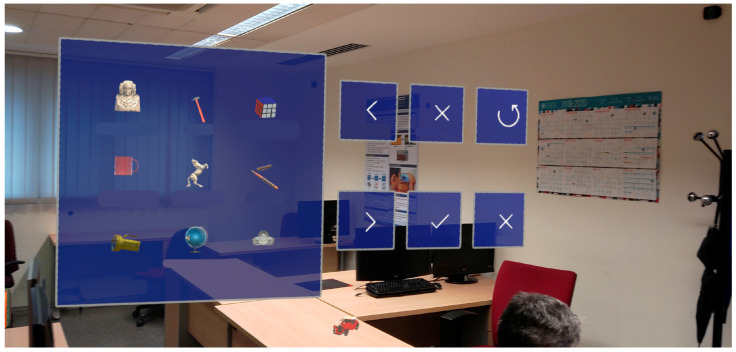
The object selection menu for setting up a task.

The main objective of the application is to allow the user to visualize virtual objects in the real world in order to remember their position and then place the objects back in the memorized locations, one by one. The setup only needs to be performed once. The application scans the environment and stores its features using the device’s sensors and the Microsoft Azure Spatial Anchors online service. The supervisor can select any number of objects from a list of pre-designed objects and place them in the physical environment. The application allows virtual objects to be placed on flat surfaces such as tables. Different configurations can be saved with their respective objects and rooms. Figure 1 shows the object selection menu for configuring a task.

The task for the participants consists of three phases: a familiarization phase, a learning phase, and an evaluation phase.

The familiarization phase: In this phase, the participants become familiar with the operation of the HoloLens 2 headset and the MR application.

The learning phase: In this phase, the participants explore a real environment, a 38 m^2^ room. They have to find virtual 3D objects that are scattered around the room. The participants can virtually touch each object and remember its location. When all of the objects have been touched, the phase ends. The time taken during this phase is recorded.

The evaluation phase: In this phase, the participants can see each virtual object, one by one, next to the starting point of the room. They place these virtual objects in the location where they think they saw them in the previous phase. Eight different 3D virtual objects were used: a violin, a set of books, a mug, an earth globe, a frog-shaped decorative object, a toy car, a camera, and a mushroom-shaped decorative object. This choice was based on previous research [43] which identified eight objects as optimal for a reliable measure of visuospatial memory in young, healthy adults. The application is designed to keep the orientation of the virtual objects perpendicular to the detected surface plane, but they can be rotated at any angle around the vertical axis.

*Hardware and software*

The headset used was the Microsoft HoloLens 2. It is an optical see-through headset with a transparent viewer on which the digital elements that the user perceives are displayed as integrated in the real environment. It is the second version of this headset manufactured by Microsoft with an improved processor, gesture recognition and iris tracking, 8 Gb of RAM instead of 2 Gb, and a larger field of view. HoloLens 2 has four visible light cameras, two infrared cameras, a depth sensor, an accelerometer, a gyroscope, a magnetometer, and omnidirectional microphones for ambient sensing. HoloLens 2 runs on a modified version of the Microsoft Windows Operating System called Windows Holographic Operating System, which was specifically designed for the headset. The version used in this work was 22H2 (Build 22621.1376).

The application was developed using Unity, which is a cross-platform 2D and 3D graphics engine [44]. Unity facilitates the development of applications in three dimensions and integrates specific libraries to deal with VR, AR, and MR. The language used was C#. The Microsoft Mixed Reality Toolkit 2 (MRTK 2) was used to develop the MR application. The MRTK is the SDK provided by Microsoft for developing applications for HoloLens [45]. For the configuration phase, the Microsoft Azure Spatial Anchors service (version 2.13) [46] was used, which allows the application to detect space through the device’s camera and depth sensors and store this information on Microsoft servers.

*MR task*

**Figure 2:**
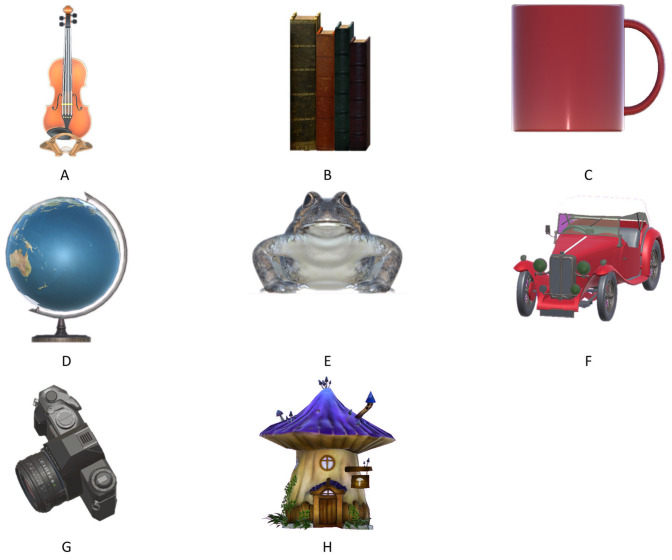
Selected 3D objects for the MR application: (**A**) a violin; (**B**) a set of books; (**C**) a mug; (**D**) an earth globe; (**E**) a frog-shaped decorative object; (**F**) a toy car; (**G**) a camera; and (**H**) a mushroom-shaped decorative object.

**Figure 3:**
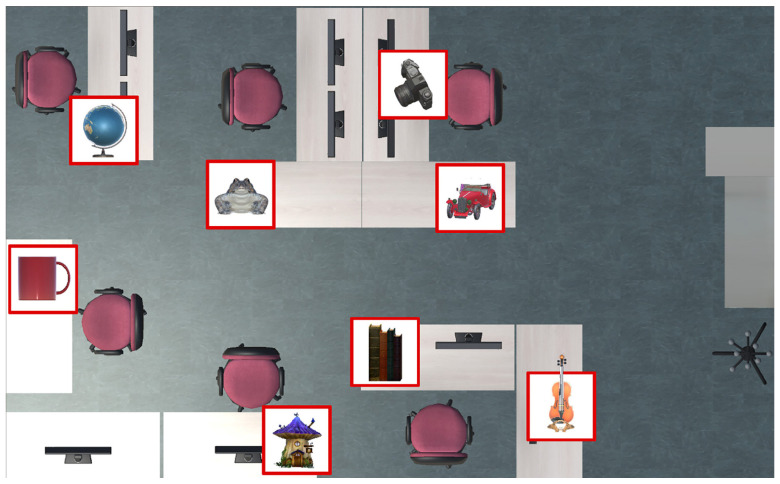
Room layout and object positions used in the MR study. The objects are highlighted with red-bordered squares.

The participants’ task was to remember the positions of eight virtual objects placed in a real environment and to place them in the remembered locations at a later phase. The objects included in this study and their positions were previously determined by the supervisor. The eight objects selected for this study are shown in Figure 2. The locations of the objects were the same for each participant and are shown in Figure 3.

**Figure 4:**
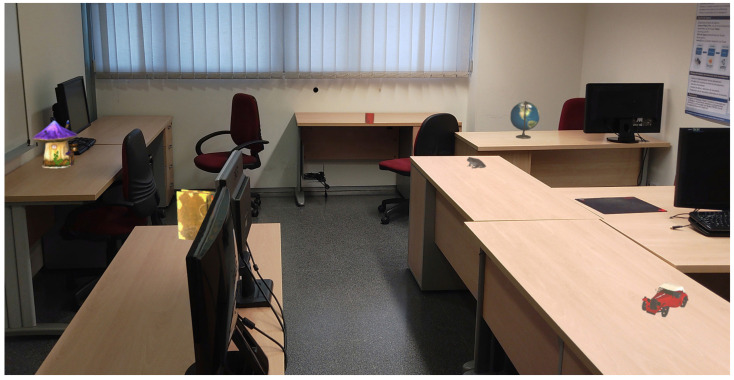
Virtual objects in the physical environment as seen by the participant.

The phases that the participants must complete are as follows:

(1) The familiarization phase: The first phase consists of placing three virtual objects on three markers that appear to be positioned in the real world. The objective of this phase is to familiarize the participants with the operation of the HoloLens 2 headset and the MR application. No data are collected during this phase.
(2) The learning phase: In this phase, the participants can visualize eight virtual objects that are located in the real world. The participants are informed that they can take as much time as they need to memorize the position of each object. When the participants think that they can remember the position of an object, they must touch it with their hand, and the object lights up. When all eight objects have been touched, the time is recorded, and the phase ends. Figure 4 shows the participants’ point of view of the room with the eight objects to be memorized.
(3) The evaluation phase: In this phase, a previously memorized object appears next to the participant (the starting area). The participant must pick up the object and place it in the location where he or she remembered it from the learning phase. If the object is placed correctly, a new object appears in the starting area of the room, and this action must be repeated until all eight objects have been placed. If the participant places an object in the wrong position, the application informs the participant with a sound. The participant is allowed up to three attempts. If all three attempts fail, the application moves on to the next object. The successes, attempts, and total time spent are automatically recorded by the application.

#### 2.3.2. Map Task

In this task, the participants are presented with a list of images of the objects labeled with letters from A to H. They are asked to mark the original position where they remember each object with its corresponding letter on a 2D map of the room using a paper format. They are asked to perform this without looking at the physical room and looking only at the map.

#### 2.3.3. Questionnaires

The participants fill out an online questionnaire with 75 questions that were grouped into the 15 variables mentioned above.

## 3. Results

This section presents the data obtained from this study and the statistical analyses performed. The Shapiro–Wilk tests indicated that the data did not follow a normal distribution. Therefore, non-parametric tests were applied. In the tables, a descriptor of each group is presented in the format (median (Mdn); interquartile range (IQR)). All of the tests are presented in the format (statistic U, normal approximation Z, *p*-value, r effect size). The results obtained were considered to be statistically significant when *p* < 0.05 and are shown in bold. The software used for the analyses was R (version 4.4.2) [47].

### 3.1. Performance Variables

**Figure 5:**
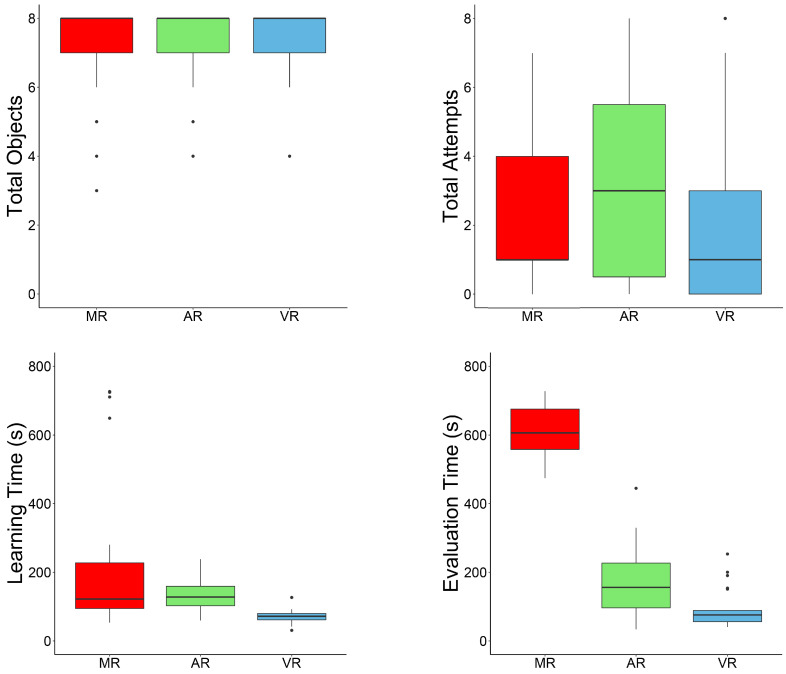
Box plots comparing the performance variables for the MR (red), AR (green), and VR (blue) applications.

Figure 5 shows box plots comparing the performance variables for the MR application with HoloLens 2, AR on a mobile device, and VR using a headset.

The Mann–Whitney U test was applied to the performance variables to determine if there were statistically significant differences. The results are presented in Table 1 and Table 2. Table 1 considers the study with the MR application using HoloLens 2 and the AR application using a Lenovo mobile device. Table 2 considers the study with the MR application using HoloLens 2 and the VR application. The Evaluation Time was significantly longer for the MR application than for the AR and VR applications. The Learning Time was significantly longer for the MR application than for the VR application. These results can be explained by the novelty of HoloLens 2 and the mechanics of each application and are discussed in the Section 4. There were no significant differences in the number of correctly placed objects and attempts between the performances of the MR application and those of the AR and VR applications.

Table 3 shows the result of the Wilcoxon signed-rank test to determine if there was a statistically significant difference between the number of objects correctly placed using the MR application and the number of objects participants placed in the map task. The result indicates that there was no statistically significant difference.

### 3.2. Gender

**Figure 6:**
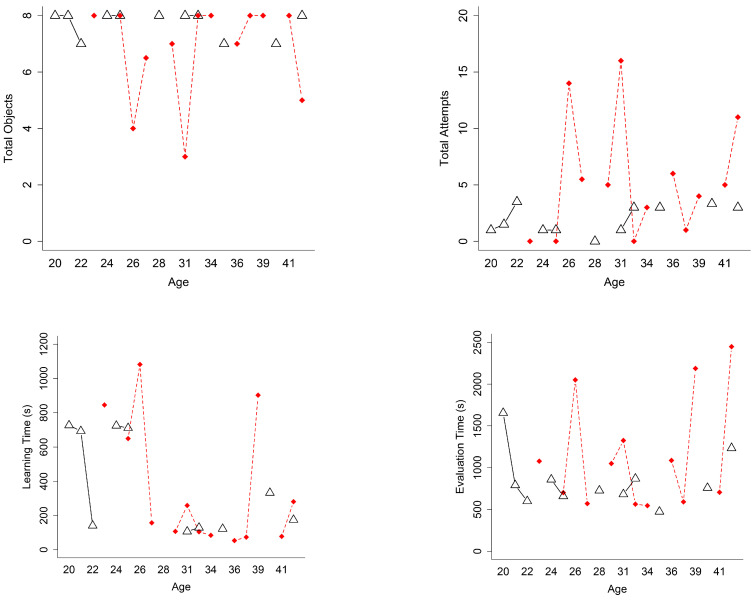
Interaction plots for the performance variables when using the MR application and taking into account the age and gender of the participants. The triangles represent the women’s group, and the red rhombuses represent the men’s group. Black solid lines connect women, while red dashed lines connect men. Lines connect only consecutive points within the same group to highlight trends and avoid overlaps.

To test whether there were significant differences in the performance variables according to the gender of the participants, a Mann–Whitney U test was applied, dividing the group into two subgroups according to gender. Table 4 shows the results of the test for the performance variables of the two subgroups (women and men). It can be observed that no statistically significant differences were found for any of the performance variables based on the gender of the participants. The interaction graphs for the performance variables are shown in Figure 6, taking into account the age and gender of the participants.

### 3.3. Subjective Variables

**Figure 7:**
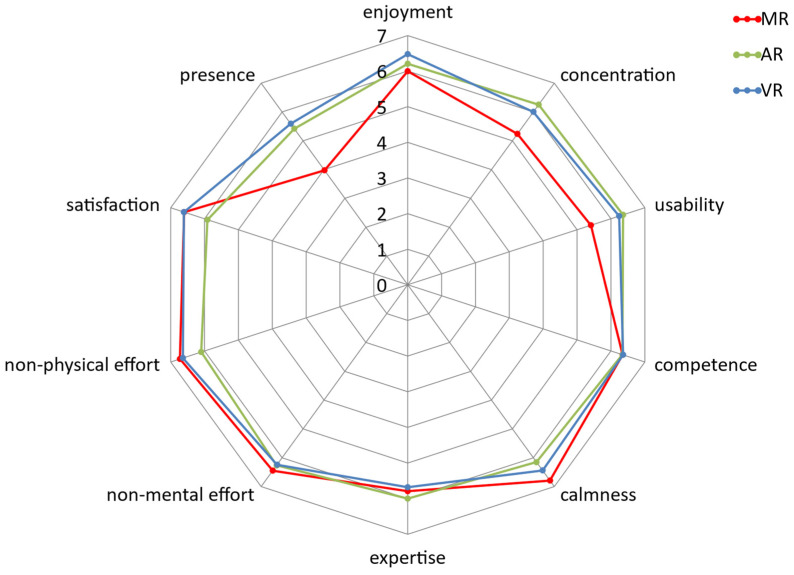
Radial graph showing the mean of the subjective variables considering the MR, AR, and VR applications.

To test whether there were statistically significant differences for the subjective variables, the data obtained using the MR application were compared with those obtained using the AR and VR applications. Mann–Whitney U tests were used for this purpose. The results of the test between the MR and AR applications are shown in Table 5. The variable of no cybersickness was excluded from this analysis due to the physical differences between the mobile device and HoloLens 2. Statistically significant differences were found for the variables of concentration, usability, calmness, non-physical effort, satisfaction, and presence. The participants experienced greater satisfaction and required less physical effort when using the MR application. Table 6 shows the results of the Mann–Whitney U test for the subjective variables when using the MR and VR applications. Statistically significant differences were found for the variables of enjoyment, usability, and presence. These significant differences can be clearly seen visually in the radial graph shown in Figure 7.

The Mann–Whitney U test was applied to the two subgroups separated by gender for the MR application to determine if there were statistically significant differences between men and women for the subjective variables. Table 7 shows the results. It can be seen that only the concentration variable showed a significant difference, with women requiring more concentration.

**Figure 8:**
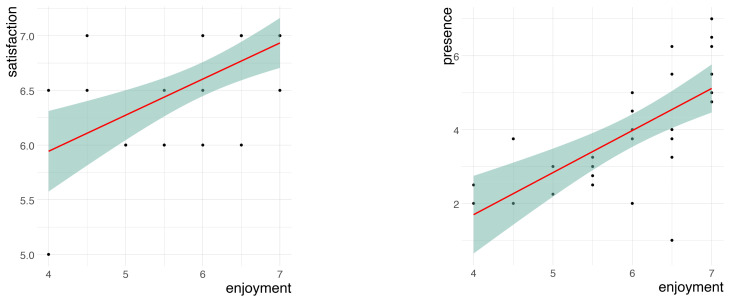
Scatter plots for the positive correlations between satisfaction and enjoyment and presence and enjoyment. The green area represents a 95% confidence level interval for predictions from a linear model. The red line represents the fitted regression line, showing the linear relationship between variables.

A Spearman correlation test was used to check the possible relationships between the subjective variables for the MR application. The results are presented in Table 8. It can be observed that there were significant correlations between the perception of concentration and the perceived enjoyment, usability, competence, expertise, satisfaction, and presence. The participants who rated usability positively perceived more enjoyment, concentration, competence, expertise, and satisfaction. The relationships between satisfaction and enjoyment and presence and enjoyment are shown visually in the scatter plot in Figure 8.

### 3.4. Relationships Between the Rest of the Subjective Variables and the Variables of Experience with Computers and Video Games

**Figure 9:**
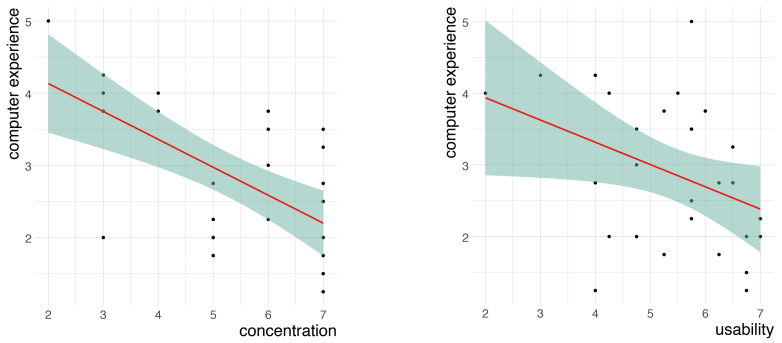
Scatter plots for the negative correlation between concentration and computer experience (**left**) and the negative correlation between usability and computer experience (**right**). The green area represents a 95% confidence level interval for predictions from a linear model. The red line represents the fitted regression line, showing the linear relationship between variables.

Table 9 shows the relationship among the rest of the subjective variables and the variable of experience with computers and video games. The video game experience is divided into five groups according to genre, as indicated previously. Significant inverse relationships were shown between computer experience and the feeling of concentration and usability during the task with the MR application. There was also an inverse relationship between the experience with video games and concentration. If we analyze the games by typology, the participants who were more accustomed to role-playing games perceived a lower sense of usability and concentration. Figure 9 shows the relationships between concentration and computer experience (left) and between usability and computer experience (right).

## 4. Discussion

In this work, an MR application using an optical see-through headset was developed for the assessment of short-term spatial memory. The application works in any indoor environment of any size. It does not require the addition of physical elements for tracking. The user has freedom of movement. Interaction is gestural and natural. The supervisor can personalize the task with the number and position of the objects to be memorized, and this configuration is stored so that the task can be repeated at any time. To our knowledge, this is the first application using an MR headset (HoloLens 2) for this purpose and with these features. In previous studies, VR headsets have already been used to explore spatial knowledge acquisition tasks in controlled environments [19,48,49]. In line with our work, which highlights the importance of VR, AR, and MR for memory-related tasks, other studies also highlight the potential of VR headsets to achieve similar objectives [19,48,49]. Additionally, like other works [19,48,49], we also evaluate the time spent on tasks and performance outcomes. Some works only use VR with headsets [19,49]. Another work compared VR with headsets and a real environment [48]. In this last work [48], a maze challenge was used as the experimental task, where participants were asked to retrieve objects placed within the maze. The participants’ performance in two conditions (VR vs. real) was compared in terms of navigation times and the routes chosen. Statistical analyses showed no significant differences between VR and real conditions in terms of total time or navigation performance, regardless of prior gaming experience or self-assessed navigation skills. The work of Monteiro et al. [48] and our work coincide in using the same environment (real or modeled) for comparisons. In our study, the participants were not instructed to complete the tasks as quickly as possible, unlike other studies which emphasized minimizing the task completion time [48]. This lack of urgency led some participants to spend extra time on certain tasks, significantly increasing their total time spent. In our work, in line with prior research [48], we analyzed whether there were correlations between the computer experience of the subjects and other study variables as well as between video game experience and other study variables. As previous studies argue [48], we agree that it is valuable to explore spatial memory using the latest VR/MR technologies, given their rapid and significant evolution, and to ensure that prior research is accessible to provide a solid foundation for future investigations. The evolution of headsets can be observed in previously published works. For example, the Oculus Rift CV1 [48], which required a computer connection to operate, the Oculus Quest [19], one of the first standalone devices, and the Meta Quest 2 [49] have already been used. In this work, HoloLens 2 is employed. To the best of our knowledge, while not specifically for spatial memory, other headsets for MR with grayscale or color passthrough have been used in various applications. For example, the Meta Quest Pro has been employed for learning to play the piano [50], and the Apple Vision Pro could open up new possibilities, such as for individuals with visual deficits [51].

Our MR application was validated with 29 participants, and the data were compared with two other studies. One of them used a mobile AR application in the same environment. The other study used a VR application, using a headset.

When comparing the MR application and the mobile AR application for the performance variables, the only difference was the Evaluation Time. The participants using the MR application took significantly more time. Our argument for this result is that the use of HoloLens 2 was new to all of the users, they enjoyed observing objects, and they had no minimum time requirement to perform the task. It is important to note that there were no significant differences for the other three performance variables.

When comparing the MR application and the VR application for the performance variables, there were differences in the Learning Time and Evaluation Time in favor of the VR application. Our explanation for this result is similar to that for the AR application: all of the users were new to using HoloLens 2, they found observing the objects appealing, and they had no minimum time requirement to perform the task.

When comparing the total number of correctly placed objects between the MR application and the map task, no statistically significant difference was found. This result indicates that the objects placed with the MR application were correctly remembered and placed on the 2D map of the environment. This result is consistent with previous works [19]. This result shows that the participants learned the spatial–visual associations and were able to transfer these associations from the 3D array of the real environment to the 2D array of the map and the mental image of the room. Thus, the first part of the main objective has been fulfilled, and the first part of the main hypothesis has been confirmed: our MR application has proven to be a useful tool for the assessment of short-term spatial memory.

The participants rated the MR experience positively, with a median rating of 6 or higher (on a scale of 1 to 7) for seven of ten subjective variables and a median rating of 7 for the satisfaction and the non-physical effort variables. Therefore, the second part of the main hypothesis has been supported: the participants had a satisfactory experience when using our MR application. No statistically significant differences were found for the performance variables when gender was taken into account. Our results are consistent with previous studies [19,39].

With regard to the comparison between the MR application and the mobile AR application for the subjective variables, there were significant differences in favor of the MR application for the variables calmness, non-physical effort, and satisfaction. Our argument for the lower perceived physical effort in the MR application is that, in the AR application, the weight of the mobile phone and its handling significantly influenced this result.

With regard to the comparison between the MR application and the mobile AR application for the subjective variables, there were significant differences for the variables concentration, usability, and sense of presence, in favor of the AR application. Our argument for the higher perceived usability in the AR application is that users are very accustomed to using a mobile phone, and it was the first time they had to use air gestures to interact with an MR application. Our argument for the lower sense of perceived sense of presence in the MR application is that the virtual objects are holograms, which are objects made of points of light. It is a different visualization than the one perceived in reality and in the rest of the visualization systems, and users perceived this difference. This is a technical limitation that will be improved in future MR headsets. One area of improvement for optical see-through headsets would be higher resolution displays. Increasing the pixel density to render sharper images with finer detail would reduce the perception of objects as mere points of light. Another option is to use the latest video see-through headsets for MR, such as Apple Vision Pro, which uses color passthrough.

After conducting this study, the advantages of MR using an optical see-through headset over mobile AR and VR using headsets were identified. Thus, the second part of the main objective has been fulfilled. First, the advantages of MR using an optical see-through headset over mobile AR are as follows:

- A more immersive experience can be created by seamlessly blending digital content with the real world.
- The freedom to interact with digital content without holding or manipulating the device allows for more natural and intuitive interactions.
- Because MR headsets project digital content directly into the user’s field of view, there are fewer environmental distractions compared to viewing content on a mobile device screen.
- Headsets are becoming more ergonomically designed, while mobile AR requires users to hold and manipulate a device.

Second, the advantages of MR with an optical see-through headset over VR with headsets are as follows:

- The environment is real and does not need to be modeled. The user’s home, a therapist’s room, or any other chosen location can be used.
- MR allows users to maintain awareness of their real environment while interacting with virtual content, increasing safety and enabling collaboration with others in physical space. In contrast, VR isolates users from the real world, which can lead to disorientation and safety issues.
- With optical see-through headsets, users can interact with virtual and physical objects using natural gestures and movements in their real environment. In VR with headsets, interaction is mainly limited to virtual objects within the modeled environment.
- MR with optical see-through headsets enables social interaction and collaboration among users in the same physical space, fostering communication and teamwork. VR with headsets tends to isolate users in individual virtual environments, limiting social interaction to online platforms.
- MR allows virtual objects to interact with real-world objects and the real world. VR with headsets lacks this capability because the virtual content is isolated from the physical world.
- MR preserves spatial cues and depth perception from the real world, allowing users to accurately perceive distances and spatial relationships between objects. In contrast, VR with headset generates spatial cues only in the virtual environment, which can lead to discrepancies between perceived and actual distances.

## 5. Conclusions

Our MR application uses HoloLens 2. It allows the subjects complete freedom of movement and natural interaction with virtual objects using their hands. The application provides high ecological validity by allowing the creation of environments that closely resemble the everyday challenges of spatial memory. The flexibility of the application allows different configurations to be set up in different environments and to be saved for standardization, thus ensuring consistent test conditions across subjects.

A study was conducted with our MR application, and the data obtained were compared with two previous studies using different technologies (AR and VR). From the results, it can be concluded that MR with HoloLens 2 is a useful tool for assessing short-term spatial memory. The use of MR was effective for the short-term memorization of the displayed objects and their position in the environment. The effectiveness was consistent with the technologies used previously (AR and VR). There were no significant differences in the MR experience based on the gender of the participants.

This work has focused on the assessment of short-term spatial memory, but a possible future application would be the study of long-term memory.

## Figures and Tables

**Table 1:** Mann–Whitney U test for the performance variables comparing the MR and AR applications.

	MR (Mdn; IQR)	AR (Mdn; IQR)	*U*	*Z*	*p*	*r*
Total Objects	8; 1	8; 1	786	0.194	0.850	0.021
Total Attempts	3; 4	3; 5	718	−0.495	0.624	0.055
Learning Time	167.65; 617.59	130.22; 63.08	962	1.877	0.061	0.207
Evaluation Time	727.38; 472.63	147.8; 125.26	1537	7.453	**<0.001**	0.823

Statistically significant results (*p* < 0.05) are shown in bold.

**Table 2:** Mann–Whitney U test for performance variables comparing the MR and VR applications.

	MR (Mdn; IQR)	VR (Mdn; IQR)	*U*	*Z*	*p*	*r*
Total Objects	8; 1	8; 1	343	−0.411	0.689	0.056
Total Attempts	3; 4	1; 5	423.5	1.079	0.284	0.147
Learning Time	167.65; 617.59	71; 18	653	5.039	**<0.001**	0.686
Evaluation Time	727.38; 472.63	412; 234	607	4.241	**<0.001**	0.577

Statistically significant results (*p* < 0.05) are shown in bold.

**Table 3:** Wilcoxon signed-rank test on the Total Objects variable and comparison of the MR application with map task successes.

MR	MAP (Mdn; IQR)	*U*	*Z*	*p*	*r*
8; 1	8; 1	4	−1.890	0.073	0.248

**Table 4:** Mann–Whitney U test for the performance variables taking into account the gender of the participants (women/men) when using the MR application.

	Women (Mdn; IQR)	Men (Mdn; IQR)	*U*	*Z*	*p*	*r*
Total Objects	8; 0	7.5; 1.75	137.5	1.676	0.099	0.311
Total Attempts	1; 2	5; 4.5	65	−1.769	0.081	0.328
Learning Time	167.65; 606.91	157.22; 467.16	123	0.786	0.451	0.146
Evaluation Time	727.38; 254.98	878.52; 670.12	88	−0.742	0.477	0.138

**Table 5:** Mann–Whitney U test for the subjective variables when comparing the MR and AR applications.

	MR (Mdn; IQR)	AR (Mdn; IQR)	*U*	*Z*	*p*	*r*
Enjoyment	6; 1.5	6; 1	589.5	−1.028	0.307	0.118
Concentration	5; 3	6.5; 1	481.0	−2.199	**0.028**	0.252
Usability	5.75; 1.5	6.66; 1	371.0	−3.345	**<0.001**	0.384
Competence	7; 1	7; 1	686.5	0.062	0.955	0.007
Calmness	7; 0	6; 2	946.0	3.290	**<0.001**	0.377
Expertise	6; 2	6; 2	590.5	−1.021	0.310	0.117
Non-mental effort	7; 1	6; 1	769.0	1.030	0.306	0.118
Non-physical effort	7; 0	6; 2	977.0	3.638	**<0.001**	0.417
Satisfaction	7; 0.5	6; 1	1056.0	4.145	**<0.001**	0.475
Presence	3.75; 2.25	5.33; 1.16	305.5	−4.024	**<0.001**	0.462

Statistically significant results (*p* < 0.05) are shown in bold.

**Table 6:** Mann–Whitney U test for the subjective variables when comparing the MR and VR applications.

	MR (Mdn; IQR)	VR (Mdn; IQR)	*U*	*Z*	*p*	*r*
No cybersickness	7; 1	7; 0	297.5	−1.665	0.099	0.227
Enjoyment	6; 1.5	7; 1	225.5	−2.529	**0.012**	0.344
Concentration	5; 3	6.5; 1.5	277.0	−1.527	0.129	0.208
Usability	5.75; 1.5	6.3; 1.3	210.5	−2.652	**0.008**	0.361
Competence	7; 1	7; 1	357.5	−0.101	0.927	0.014
Calmness	7; 0	7; 1	445.0	1.914	0.057	0.260
Expertise	6; 2	6; 2	334.5	−0.507	0.618	0.069
Non-mental effort	7; 1	7; 1	362.0	−0.010	1.000	0.001
Non-physical effort	7; 0	7; 0	369.0	0.207	0.849	0.028
Satisfaction	7; 0.5	7; 0.6	341.0	−0.407	0.691	0.055
Presence	3.75; 2.25	6; 2	156.5	−3.601	**<0.001**	0.490

Statistically significant results (*p* < 0.05) are shown in bold.

**Table 7:** Mann–Whitney U test for the subjective variables when using the MR application and taking into account the gender of the participants (women/men).

	Women (Mdn; IQR)	Men (Mdn; IQR)	*U*	*Z*	*p*	*r*
No cybersickness	7; 0	7; 2.5	123.0	0.997	0.332	0.185
Enjoyment	6.5; 1	5.75; 1.375	137.5	1.446	0.155	0.268
Concentration	6; 2	4; 2.75	158.5	2.409	**0.017**	0.447
Usability	6; 1.875	5.375; 1.5625	133.5	1.249	0.220	0.232
Competence	7; 1	7; 1	122.5	0.887	0.389	0.165
Calmness	7; 0	7; 0	121.0	1.166	0.259	0.217
Expertise	6; 1.5	5.5; 1	129.5	1.121	0.272	0.208
Non-mental effort	7; 1	7; 1	92.0	−0.638	0.540	0.118
Non-physical effort	7; 0	7; 0	99.5	−0.454	0.680	0.084
Ergonomics	7; 1	6; 1	118.5	0.642	0.536	0.119
Satisfaction	7; 0.5	6.5; 0.875	121.0	0.762	0.460	0.142
Presence	4.5; 2.37	3.5; 2.3125	105.5	0.022	1.000	0.004
Anxiety	11; 5.5	9.5; 3.75	130.5	1.119	0.273	0.208

Statistically significant results (*p* < 0.05) are shown in bold.

**Table 8:** Spearman’s correlation for the subjective variables when using the MR application.

	c2 ^2^	c3 ^3^	c4 ^4^	c5 ^5^	c6 ^6^	c7 ^7^	c8 ^8^	c9 ^9^	c10 ^10^	c11 ^11^	c12 ^12^
c1 ^1^	0.20	−0.08	0.09	0.12	0.15	0.12	−0.02	0.02	0.19	−0.03	0.18
c2 ^2^		**0.56**	**0.43**	**0.72**	0.24	**0.71**	0.32	0.21	0.35	**0.64**	**0.76**
c3 ^3^			**0.55**	**0.38**	0.18	**0.39**	0.02	0.21	0.07	**0.43**	**0.38**
c4 ^4^				**0.44**	0.15	**0.42**	0.35	0.21	0.32	**0.47**	0.16
c5 ^5^					**0.41**	**0.50**	0.29	0.14	**0.45**	**0.66**	**0.51**
c6 ^6^						**0.37**	0.18	−0.14	0.29	0.03	0.04
c7 ^7^							0.25	−0.07	0.35	**0.48**	**0.64**
c8 ^8^								**0.39**	0.34	0.22	0.22
c9 ^9^									0.31	**0.42**	0.10
c10 ^10^										**0.46**	0.15
c11 ^11^											0.33

^1^ No cybersickness; ^2^ enjoyment; ^3^ concentration; ^4^ usability; ^5^ competence; ^6^ calmness; ^7^ expertise; ^8^ non-mental effort; ^9^ non-physical effort; ^10^ ergonomics; ^11^ satisfaction; ^12^ presence. Statistically significant results (*p* < 0.05) are shown in bold.

**Table 9:** Spearman’s correlation for the subjective variables after performing the task with the MR application and the variable of experience with computers and video games.

	c1 ^1^	c2 ^2^	c3 ^3^	c4 ^4^	c5 ^5^	c6 ^6^	c7 ^7^	c8 ^8^	c9 ^9^	c10 ^10^	c11 ^11^	c12 ^12^
g1 ^g1^	−0.01	−0.19	**−0.49**	−0.13	−0.09	0.20	−0.01	0.19	−0.13	−0.30	−0.15	−0.19
g2 ^g2^	−0.11	−0.28	**−0.52**	−0.29	−0.04	−0.07	−0.05	0.14	−0.11	−0.06	0.03	−0.16
g3 ^g3^	0.04	−0.07	−0.21	−0.05	0.07	0.02	0.04	0.18	0.19	−0.16	0.09	−0.07
g4 ^g4^	−0.08	−0.14	−0.19	−0.25	−0.01	0.03	0.11	−0.16	0.00	−0.27	0.21	−0.17
g5 ^g5^	−0.04	−0.04	**−0.51**	**−0.38**	−0.04	0.06	0.20	0.14	−0.01	−0.01	0.00	0.02
gt ^gt^	−0.12	−0.16	**−0.53**	−0.31	−0.02	0.13	0.14	0.04	−0.09	−0.19	0.01	−0.09
e ^e^	−0.02	−0.27	**−0.60**	**−0.41**	−0.18	0.21	0.12	−0.16	−0.22	−0.03	−0.15	−0.20

^1^ No cybersickness; ^2^ enjoyment; ^3^ concentration; ^4^ usability; ^5^ competence; ^6^ calmness; ^7^ expertise; ^8^ non-mental effort; ^9^ non-physical effort; ^10^ ergonomics; ^11^ satisfaction; ^12^ presence; ^g1^ first person; ^g2^ simulator; ^g3^ strategy; ^g4^ turn-based; ^g5^ role-playing; ^gt^ video game experience; ^e^ computer experience. Statistically significant results (*p* < 0.05) are shown in bold.

## Data Availability

The data presented in this study are available upon reasonable request from the corresponding authors.
